# Microbiota transplantation

**DOI:** 10.1016/j.heliyon.2024.e39047

**Published:** 2024-10-11

**Authors:** Javad Nezhadi, Manouchehr Fadaee, Somayeh Ahmadi, Hossein Samadi Kafil

**Affiliations:** a Student Research Committee, Tabriz University of Medical Science, Tabriz, Iran; b Department of Microbiology, Faculty of Medicine, Tabriz University of Medical Science, Tabriz, Iran; c Immunology Research Center, Faculty of Medicine, Tabriz University of Medical Science, Tabriz, Iran; d Drug Applied Research Center, Faculty of Medicine, Tabriz University of Medical Sciences, Tabriz, Iran

**Keywords:** Microbiota, Dysbiosis, MT, FMT, VMT, SMT, OMT, WMT, SiMT

## Abstract

Microbiota refers to a collection of living microorganisms, including bacteria, yeasts, and viruses, that coexist in various sites of the human body. Microbiota can perform multiple functions in the body, which have an essential effect on human health and homeostasis. For example, the microbiota can digest polysaccharides, produce vitamins, modulate the immune system, and protect the body against pathogens. Various factors can occasionally alter the microbiota population in the human body, a condition known as dysbiosis. Dysbiosis can disrupt the homeostasis of a person's body and cause disease. Recent years have witnessed efforts to restore the microbiota population of an individual's body to its original state and eradicate dysbiosis through microbiota transplantation. The noteworthy point is that different methods such as fecal microbiota transplantation, vaginal microbiota transplantation (VMT), skin microbiota transplantation (SMT), oral microbiota transplantation (OMT), washed microbiota transplantation (WMT), and sinonasal microbiota transplantation (SiMT) are used for microbiota transplantation (MT). According to the results of studies and the usefulness of MT in improving a person's health, the purpose of this study is to investigate different methods of MT to eliminate dysbiosis.

## 1 Introduction

Microbiota refers to a collection of living microorganisms, including bacteria, yeasts, and viruses, which coexist in various sites of the human body [[1], [2], [3]]. Although the terms microbiota and microbiome are often used interchangeably, they have some distinct differences. As previously stated, the microbiota is defined as living microorganisms that can be present in different body parts, such as the mouth, skin, and gastrointestinal tract. In return, the term microbiome contains both the microbiota and structural elements, metabolites, and the surrounding environmental conditions [[4], [5], [6]]. Microbiota can perform various functions in the body that have an essential effect on human health and homeostasis. For example, the microbiota can digest polysaccharides, the production of vitamins, the modulation of the immune system, and protect the body against pathogens [[7], [8], [9], [10]]. However, stress, lifestyle, diet, and the use of drugs (antibiotics) can sometimes lead to changes in the microbiota population in the human body, a condition known as dysbiosis [[11], [12], [13], [14]]. Dysbiosis can disrupt the homeostasis of a person's body and cause disease [[15], [16], [17]]. Considering the importance and different roles of microbiota in human health in recent years, efforts have been made to return the microbiota population of the individual's body to its original state and eliminate dysbiosis by using microbiota transplantation (MT) [18,19]. It is noteworthy that various methods such as fecal microbiota transplantation (FMT), vaginal microbiota transplantation (VMT), skin microbiota transplantation (SMT), oral microbiota transplantation (OMT), washed microbiota transplantation (WMT), and sinonasal microbiota transplantation (SiMT) are used for microbiota transplantation (MT). Studies have determined that MT can significantly contribute to the elimination of dysbiosis and the restoration of a person's health [[20], [21], [22], [23], [24], [25], [26]]. Based on the findings of previous research and the demonstrated benefits of MT in enhancing human health, this study aims to explore various MT techniques for the elimination of dysbiosis.

## 2 Method

In this narrative-review article, we reviewed articles and studies on microbiota transplantation to cure dysbiosis and treat patients using the PubMed, Scopus, Embase, Google Scholar and Web of Science databases.

## 3 Different methods of MT

### 3.1 Fecal microbiota transplantation (FMT)

**Fig. 1:**
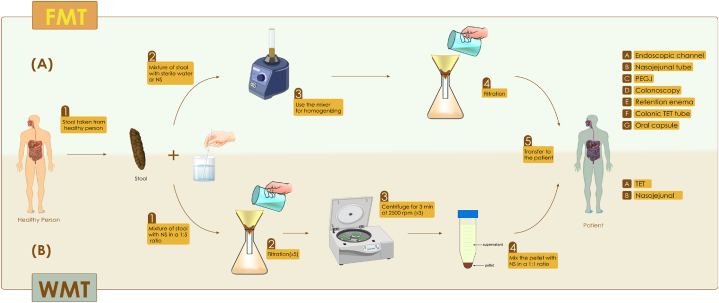
A. Stool preparation and methods of transferring FMT to the patient; B. Modified stool preparation in WMT and methods of transferring WMT to the patient with *H.pylori* infection. NS: Normal saline.

FMT transfers a solution of fecal matter from a healthy donor to the intestinal tract of a patient to eliminate dysbiosis and improve the patient's condition [27,28]. Ge Hong proposed FMT as a treatment method in China in the 4th century, using it to treat various diseases, including diarrhea [29]. In 1958, Eiseman and colleagues mentioned FMT as a treatment method for pseudomembranous colitis, and FMT found a way to enter mainstream medicine [30]. First, participants who are willing to donate stool are chosen; donors shouldn't have a family history of autoimmune, metabolic, or malignant diseases. They are also screened for any possible pathogens. Next, the donated poop is mixed with normal saline or water and made ready after being filtered to get rid of particles [[31], [32], [33]]. The stool that has been decomposed and prepared can then be given through the upper digestive tract (including the endoscopic channel, nasojejunal, or percutaneous endoscopic gastrostomy with jejunal extension) or the lower digestive tract (including colonoscopy, retention enema, colonic transendoscopic enteral tubing (TET)), or it can be given in capsule form (Table 1, Fig. 1-A) [34,35]. Today, FMT can be used to treat several gastrointestinal disorders, such as inflammatory bowel disease (IBD), Crohn's disease (CD), and *Clostridium difficile* infection (CDI) and recurrent CDI [[36], [37], [38], [39]]. Besides intestinal disorders, FMT also treats neurological disorders like Alzheimer's, Parkinson's, and Multiple Sclerosis (MS) [[40], [41], [42]]. Also, studies show that in addition to the disease mentioned above, FMT can play an influential role in treating metabolic disorders (obesity) and cancer [41,43,44]. In addition to the mentioned diseases, current research is exploring the effectiveness of FMT in treating various diseases, such as asthma, pouchitis, microscopic colitis (MC), psoriasis, epilepsy, and acute pancreatitis [43,45,46]. However, given its significant role in treating various diseases, FMT, like other treatment methods, carries risks for the recipient; incorrect donor screening could lead to the recipient contracting infectious diseases like hepatitis or HIV. Also, during the mechanical transfer of the feces by the methods mentioned above (nasojejunal, colonoscopy, or retention enema), the recipient's organs may be damaged [[47], [48], [49]].

**Table 1:** Different methods of delivering FMT to the patient.

Method of delivery	Advantages	Limitation
Endoscopic channel	Easy to deliver	Only used during colonoscopy Challenging to hold the infused suspension in the colon Not convenient to repeat
Nasojejunal tube	Easy to deliver Easy to maintain	Only used in patients with nasojejunal tube Bacteria possibly affected by bile salts Potential risk of SIBO
PEGJ	Easy to deliver Easy to maintain	Only used in few patients with PEGJ tube Bacteria possibly affected by bile salts Potential risk of SIBO
Colonoscopy	Strong evidence of efficacy for rDCI	Necessity for sedation Necessity for technical expertise Additional cost
Retention enema	Low cost Well tolerated	Inability to reach the right-side colon Modality with the lowest efficacy
Colonic TET tube	Convenient to repeat FMT Easy to deliver Avoiding bacteria affected by bile salts Easy to maintain	TET tube must be placed under colonoscopy
Oral capsules	Noninvasive Cost and time saving Convenience of administration	A large burden of the capsule Risk of vomiting and aspiration

PEGJ: percutaneous endoscopic gastrostomy with jejunal extension; SIBO: small intestinal bacterial overgrowth; rDCI: recurrent Clostridium difficile infection; TET: transendoscopic enteral tubing.

### 3.2 Vaginal microbiota transplantation (VMT)

**Fig. 2:**
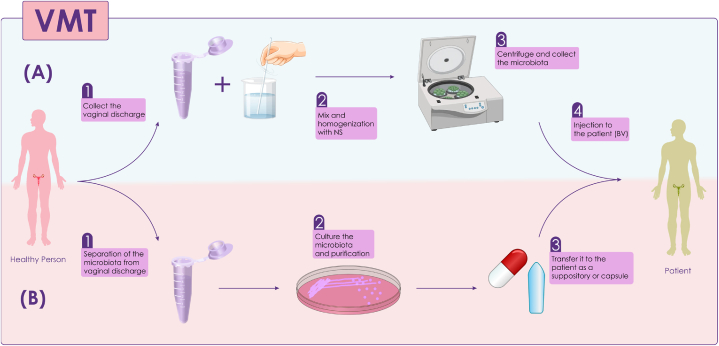
A. Direct transmission of vaginal secretions from a healthy person to a person with BV; B. Culture and purification of microbiota and transfer to a patient with BV as a suppository or capsule. NS: Normal Saline; BV: Bacterial Vaginosis.

As previously mentioned, FMT has the potential to effectively treat recurrent *Clostridium difficile* Colitis [50,51]. FMT's success in treating colitis has drawn attention to other methods of MT, including VMT. VMT involves the transfer of vaginal fluid from a healthy individual with high Lactobacillus abundance to a patient [[52], [53], [54]]. Similar to FMT, it is recommended that the donor undergo stringent screening tests before VMT to minimize potential side effects. Several tests are used for screening, such as syphilis tests (Trep-Sure), HIV 1 and 2 antibodies and antigens, HIV viral load, Hepatitis A IgM, Hepatitis B surface antigen, Hepatitis B core antibody, Total and IgM, Hepatitis C antibody, and human T-lymphotrophic virus types 1 and 2 [52,55]. There are two different methods to perform VMT. The first method collects the vaginal fluid in a disposable cup, mixes it with sterile normal saline, homogenizes it, and then collects the vaginal microbiota using a high-speed centrifuge and finally, the vaginal microbiota is directly injected into the vagina of a patient. The second method involves isolating the microbiota from the donor's vaginal secretions, cultivating and purifying it in a nutrient medium, and then transplanting it into the bacterial vaginosis (BV) patient as capsules or suppositories [21] (Fig. 2). Research reviews and studies have revealed that VMT could serve as a novel treatment for BV [56]. BV is a syndrome in which the patient has a foul-smelling vaginal discharge that affects 30 % of women worldwide [57]. BV occurs when an overgrowth of certain bacteria in the vagina disrupts the natural balance of the vaginal microbiota. Also, BV can increase the chance of getting a sexually transmitted disease (STD), including HIV 2, and reproductive outcomes such as cervical dysplasia, miscarriage, and preterm birth [58]. Antibiotics like metronidazole typically treat BV. While this antibiotic reduces the total number of BV-associated microbes and temporarily alleviates symptoms, 30–60 % of patients typically experience a recurrence of BV one month after antibiotic therapy [57,59,60]. In 2019, researchers recorded the first report on the positive role of VMT in treating BV. This report revealed that 4 out of 5 women who underwent two years of antibiotic treatment and then received a small amount of fresh vaginal fluid recovered from BV. Although this study identified the positive role of VMT in treating BV and reported no severe side effects, the small study size and lack of a placebo complicate the interpretation of whether VMT alone offers additional benefits over antibiotics [52]. Researchers are currently investigating the effectiveness of VMT in other diseases, including ovarian cancer, recurrent miscarriage, and endometrial diseases [61,62]. However, like FMT, VMT can cause complications for the recipient, and improper screening can lead to infectious diseases. So examining the different aspects of VMT requires more studies and clinical trials [56,63].

### 3.3 Skin microbiota transplantation (SMT)

**Fig. 3:**
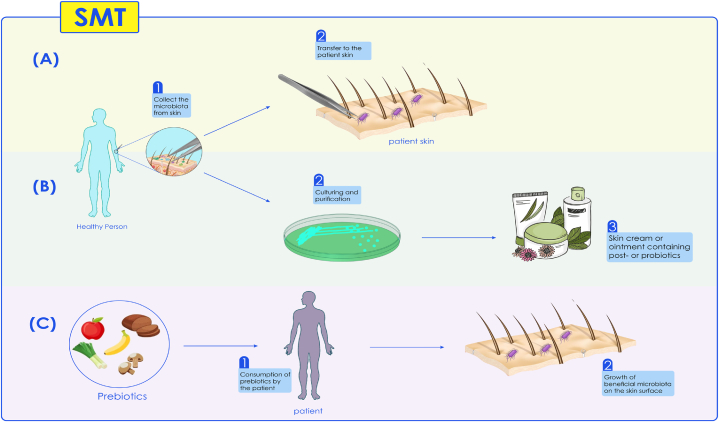
A. Direct transfer of microbiota from the skin of a healthy person to a particular area of the skin of a patient; B. Culture and purification of microbiota and use as pro and postbiotics in cosmetics and hygiene; C. Feeding the patients with foods containing prebiotics to increase the growth of beneficial microbiota in the patient's skin.

As a first line of defense, the skin plays a vital role in resisting infectious agents. The skin plays a role against infectious agents through various protective mechanisms, including acidic pH (acid mantle). Recent research has revealed that the skin's acidic pH is not the sole factor contributing to its resistance to infections. The presence of healthy microbiota colonizing the skin also plays a significant role in supporting the body's defenses against infections through various mechanisms [[64], [65], [66]]. Occasionally, a disturbance in the balance of the skin's microbial population can lead to skin disorders [67]. SMT is one of the methods that has received attention in recent years for treating dysbiosis [68]. SMT transfers helpful skin microbiota from a healthy person to a patient with skin disease. Similar to the previously mentioned methods, SMT involves screening and monitoring the donor for skin and infectious diseases [22,69]. There are three strategies for manipulating the skin's microbiota for therapeutic purposes. In the first strategy, the beneficial microbiota of the skin is collected from a healthy person and transferred directly to a specific area of the patient's skin. Before transfer, the patient must wash and disinfect the desired area of their skin. The first strategy is known as skin microbiota transplantation [70]. In the second strategy, beneficial microbiota is first received from a healthy person. Then this microbiota is grown in a pure form in the culture medium and transferred to the skin of a patient to improve health. In this strategy, like the first strategy, a specific part of the patient's skin must be washed and disinfected beforehand. These live microbiota are called probiotics (probiotics are live microorganisms that are intended to have health benefits when consumed or applied to the body) [70,71]. In the second strategy, in addition to probiotics, postbiotics can also be used as a therapeutic aim, in which the products produced by bacteria, including enzymes, antioxidants, amino acids, lipids, and vitamins, are collected purified, and transferred to the patient. In the third strategy, the microbiota in the patient's skin is changed by using prebiotics. In the third strategy, the microbiota in the patient's skin is altered by using prebiotics. In this process, prebiotics are first given to the patient to cause the growth of specific microbiota on the surface of the skin, and microbiota can play a positive role in promoting skin health. Prebiotics are an ingredient with a bio-selective activity that acts as food for human microbiota. Prebiotics are used to improve the balance of these microorganisms (Table 2, Fig. 3) [67,72,73]. Various research reviews and studies have revealed that SMT can serve as a novel treatment method for *Malassezia furfur* infections [74]. Usually found on the skin surfaces of humans and all kinds of animals, *M. furfur* belongs to the monophyletic genus of fungi. This yeast is dependent on lipids and typically accounts for more than 80 % of the fungal population on the surface of human skin. It is isolated in both healthy and diseased hosts [75,76]. *Malassezia* spp. can be involved in several common dermatologic disorders, such as Seborrheic Dermatitis (SD), Pityriasis Versicolor (PV), and Malassezia folliculitis [77]. Also, studies show that SMT can be effective as a therapeutic method to treat axillary osmidrosis [78]. However, examining the different aspects of SMT requires more studies and clinical trials [22].

**Table 2:** Advantages and disadvantages of different strategies in SMT.

Strategies	Advantages	Disadvantages	References
1) Skin microbiota transplant	a) The microbiota is transferred In its natural environment	a) Only a low number of bacteria can be harvested from a person's skin b) A culturing step is typically necessary sufficient amounts of bacteria c) This method is not scalable or industry-applicable	[36,70]
2) Skin bacteriotherapy	a) The process is easily scalable and thus industrial applicable b) Pro- or postbiotics can be applied in a skin emollient, crème or suitable medium for skin	a) Bacteria are cultured in sugar-rich media; it can, therefore be more difficult for the bacteria to adjust to a sebum-rich environment b) Skin engraftment is not easy; the applied bacteria compete with the skin resident microbiota of the deeper skin layers c) The application of high amounts of bacteria could lead to a skin-immune reaction with irritation and side effects as a result	[96]
3) Prebiotic stimulation	a) There is no need to work with living bacteria; thus, there is a reduced chance of a skin immune reaction	a) Prebiotics could stimulate non-targeted low-abundance bacteria b) The effect of prebiotics can be unpredictable given the variability in the, skin microbiota, physiology, and immune response in different individuals	[72]

### 3.4 Oral microbiota transplantation (OMT)

**Fig. 4:**
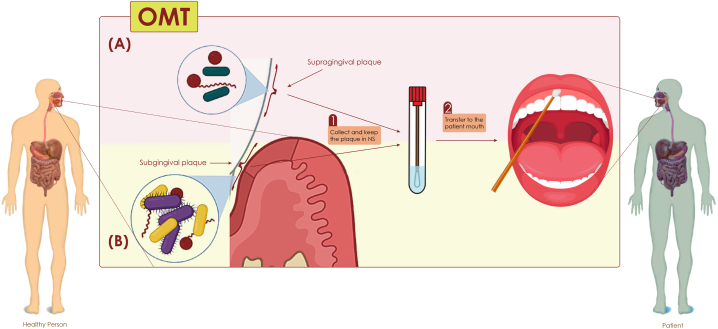
A. Collecting the microbiota of the supragingival and transferring it to the patient using a nylon swab; B. Collecting the microbiota of the supragingival or subgingival and transferring it to the patient (hypothetical methods of OMT). NS: Normal Saline.

Involuntary transmission of oral microorganisms with saliva from one person to another is one of the common events in life [79,80]. In contrast, OMT is not part of a natural event and is used to cure diseases related to periodontitis and caries, in which oral biofilms are transferred from a healthy donor to a patient. While no actual OMT has been reported to date, this concept has been proposed hypothetically by researchers such as Floyd Dewhirst, Diane Hoffmann, and others [81,82]. Dewhirst and Hoffmann suggest collecting supragingival plaques from a healthy donor without caries as the first step in their OMT hypothesis. In the next step, after collecting plaques from healthy donors, the collected plaques are stored in a saline solution, and a nylon swab is used to transfer them to a patient with active caries. According to the protocol suggested by these researchers, the donor must have a healthy oral microbiota, which can destroy bacteria that cause tooth decay, including streptococcus mutans (Fig. 4-A). In a different study, Pozhitkov and colleagues proposed the introduction of beneficial microbiota into the oral cavity of patients suffering from periodontitis [82]. First, Pozhitkov and colleagues determined that the microbiota population in healthy individuals is different from that in individuals with periodontitis. They then designed an in vitro antimicrobial protocol before OMT. They demonstrated that using sodium hypochlorite (NaOCl), followed by its neutralization with sodium ascorbate buffer, could contribute to the elimination of periodontitis-associated microbiota and allow for a change in the microbial population after OMT. In this study, the first stage of OMT involved collecting sub- and supra-gingival plaque from healthy donors, preferably the patient's wife. The second stage involved deeply washing, cleaning, and treating the roots of a periodontitis patient's teeth with a broad-spectrum antibacterial agent. In the final stage, the mouth of a patient suffering from periodontitis is washed with a microbial suspension prepared from a healthy donor to replace the beneficial microbiota (Fig. 4-B). Safety concerns dependent on the potential application of OMT are similar to those for oral probiotics [82,83]. Transplanted plaques, like probiotics, should not cause disease and should have a high degree of genetic stability. At this point in the research, the optimal approach for OMT has not yet been determined. Key questions remain unanswered, such as whether oral plaques should be transplanted directly from a healthy donor to the patient, whether pathogenic microorganisms within the plaque should be eliminated before transplantation, or whether the plaque should be cultivated under in vitro conditions, combined with commensal microorganisms [84,85]. Other issues at this stage of research are still being debated, including whether the patient's oral cavity should be disinfected before the OMT or not, whether a single dose of OMT is sufficient for effective and permanent colonization of beneficial microbiota, and whether higher doses of OMT are required [82]. Finally, according to the studies conducted on OMT, it can be noted that OMT can be a new method for treating and managing caries and periodontitis. However, at this stage, clinical recommendations for using OMT cannot be given due to the limited and few studies [23].

### 3.5 Washed microbiota transplantation (WMT)

WMT is considered a subset of FMT, drawing its foundation from this method. Research has proven that using WMT not only significantly reduces the side effects of creating a stool suspension using traditional methods (the stool preparation method in FMT), but also significantly improves efficiency [[86], [87], [88]]. Nowadays, it is thought that WMT alone or in combination with the currently recommended regimen can be a new strategy to eradicate Helicobacter pylori infection by restoring the gut microbiota population to a normal state [24,89]. To perform WMT, all the donors had to complete a structured questionnaire, and after having the conditions, they had to participate in physical and psychological examinations. The study excluded all donors suffering from gastrointestinal diseases, infectious diseases, metabolic diseases, allergic diseases, autoimmune diseases, chronic fatigue syndrome, and even neurological disorders. Also, all eligible donors received the necessary training about a healthy diet before donating stool [24,90,91]. In the WMT first, the stool sample taken from healthy donors is collected and weighed, then the stool is added to sterile saline based on the ratio of stool to saline (1:5), and then the combination of stool and saline is mixed by using a blender; the mixture is filtered through the intelligent microbial separation system ( × 5), then the resulting suspension is immediately centrifuged at 2500 rpm for 3 min ( × 3), and finally, the sediment obtained after centrifugation is resuspended in sterile saline (1:1). It is advised to administer a proton pump inhibitor, such as omeprazole or lansoprazole, intravenously 1 h before WMT, along with an intramuscular dose of metoclopramide to the recipient 30 min before the WMT [24,91]. A nasojejunal or TET is used to transfer the prepared suspension from the donor to the recipient (Fig. 1-B) [30]. In WMT, 200 ml of prepared fecal suspension is injected into the recipient, and the patient was instructed to remain in a lying position for 30 min while avoiding any strenuous physical activity. Typically, the physician performs WMT once a day for three consecutive days, depending on the patient's condition [24]. Currently, researchers have determined that WMT can effectively treat infections caused by H. pylori. Therefore, due to WMT's impact on H. pylori, this method has the potential to treat various stomach-related ailments in the future, offering a fresh approach to treating other stomach-related issues [24]. However, more clinical studies are required to confirm the efficacy, as well as safety, of WMT, especially in combination with currently recommended regimens in randomized controlled trials.

### 3.6 Sinonasal microbiota transplantation (SiMT)

**Fig. 5:**
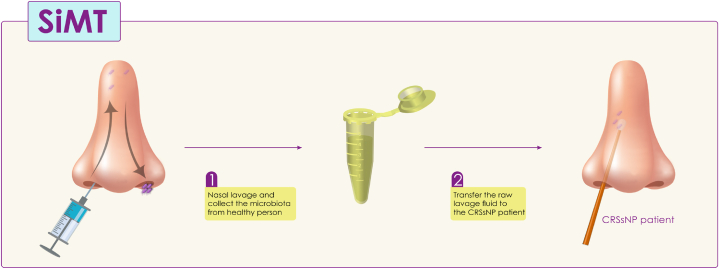
Collection of beneficial nasal microbiota from a healthy person and transferring the nasal discharge (raw lavage fluid) to a patient with CRSsNP. CRSsNP: Chronic or recurrent rhinosinusitis without nasal polyps.

In the SiMT, like the other methods mentioned, transfers beneficial microbiota from a healthy person to a patient. To perform SiMT, similar to other forms of MT, donors are screened for infectious and other diseases to reduce risk to recipients [25,92]. After screening, the patients were treated with antibiotics to reduce the bacterial load of the nose and paranasal sinuses. After antibiotic therapy, healthy microbiota is collected from donors by nasal lavage (raw lavage fluid is commonly used for MT), and then the raw lavage fluid of healthy people is transferred to the patient; this process is usually repeated for five consecutive days (Fig. 5) [25,93]. Research has demonstrated that transplanting nasal microbiota from healthy individuals to patients can alleviate the symptoms of chronic or recurrent rhinosinusitis without nasal polyps (CRSsNP) [25,93,94]. Patients reported a significant reduction in symptoms after SiMT, potentially due to an increase in the frequency and diversity of the nose's microbiota. These studies bolster the hypothesis that disruptions in the nose's microbiota or microbiome can trigger the inflammation associated with CRSsNP, and that restoring the microbiota's diversity and frequency in CRSsNP patients could potentially offer therapeutic benefits [25]. As a result, according to the studies conducted, it can be pointed out that transplanting nasal microbiota to patients with CRSsNP may cause a long-term change in the frequency and diversity of nasal microbiota and decreased symptoms. Furthermore, it is essential to note that SiMT, in combination with antibiotics, can be a new treatment strategy for CRSsNP in the future. Although more randomized/controlled studies are necessary for using SiMT in treating patients [[95], [96], [97]].

## 4 Conclusion

Researchers have designed numerous investigations in recent years to evaluate the effects of microbiota transplantation on people's health, leading to insights into their potential use to improve health and even treat diseases and disorders. The review of various studies revealed that the transfer of microbiota from healthy donors to patient recipients can effectively eliminate dysbiosis and improve patient condition. However, investigating different aspects of MT and each of the methods mentioned above requires more research and studies in the future.

## CRediT authorship contribution statement

**Javad Nezhadi:** Writing – review & editing, Writing – original draft, Investigation, Formal analysis, Data curation. **Manouchehr Fadaee:** Writing – review & editing, Writing – original draft, Investigation. **Somayeh Ahmadi:** Writing – review & editing, Writing – original draft, Data curation. **Hossein Samadi Kafil:** Writing – review & editing, Writing – original draft, Visualization, Supervision, Methodology, Investigation, Funding acquisition, Formal analysis, Data curation, Conceptualization.

## Ethical approval

Study was approved by local ethic committee of Tabriz University of Medical Sciences, Tabriz, Iran.

## Funding

This study was supported by 10.13039/501100021771Student Research Committee, Tabriz University of Medical Sciences under grant number 74829.

## Declaration of competing interest

The authors declare that they have no known competing financial interests or personal relationships that could have appeared to influence the work reported in this paper.
